# The effect of intra-articular extended-release triamcinolone acetonide on OARSI-recommended physical performance measures in adults with bilateral symptomatic knee osteoarthritis

**DOI:** 10.1016/j.ocarto.2022.100268

**Published:** 2022-05-26

**Authors:** N.A. Segal, J.C. Alm

**Affiliations:** Department of Rehabilitation Medicine; The University of Kansas, 3901 Rainbow Boulevard, Mailstop 1046, Kansas City, KS, 66160, USA

**Keywords:** Triamcinolone acetonide, Osteoarthritis, knee, Physical function, Pain, knee

## Abstract

**Objective:**

While the effect of triamcinolone acetonide extended-release (TA-ER) on reducing knee osteoarthritis (OA) pain has been reported, the effects on physical performance are incompletely understood. This open label clinical trial systematically evaluated the effects of intra-articular TA-ER on physical performance, self-reported function, and quality of life in participants with bilateral symptomatic knee OA 6, 12 (primary) and 24 weeks following bilateral injection of TA-ER (32 ​mg).

**Methods:**

Seventy participants were enrolled (61.4% women; age 64.0 ​± ​11.7; BMI 31.8 ​± ​5.7 ​kg/m^2^). Physical performance was measured by 30-s chair stand test, 40 ​m fast paced walk test (FPWT), and stair negotiation test at baseline and each follow-up visit. Physical function and quality of life (QOL) were measured with the Knee Injury and Osteoarthritis Outcome Score (KOOS-PS) and pain was measured with numeric rating scale (NRS).

**Results:**

In comparison with baseline, self-reported pain, function, and quality of life were improved at each follow-up through 24 weeks and the number of chair-stands significantly improved following treatment by (mean ​± ​SE) 1.9 ​± ​0.6 ​at 6-week (p ​= ​0.0048) and by 1.8 ​± ​0.5 ​at 12-week follow-up (p ​= ​0.0011) but was not statistically significant at 24-week follow-up (0.6 ​± ​0.6; p ​= ​0.4711). Stair negotiation times were 7.2 ​± ​3.7, 7.1 ​± ​3.8, and 5.4 ​± ​4.0 ​s lower at the three respective follow-up timepoints, although these changes did not reach statistical significance (p ​= ​0.0530, p ​= ​0.0599, and p ​= ​0.1793 respectively). The 40m-FPWT time did not significantly improve.

**Conclusion:**

These data indicate improvement in chair stand performance through 12 weeks post-injection and sustained improvement in participant-reported physical function through 24-week follow-up in adults with bilateral painful knee OA treated with TA-ER.

## 1 Introduction

Non-surgical treatments for knee OA have traditionally consisted of a combination of temporizing measures such as bracing, non-pharmacological modalities, physical therapy and topical, oral and intra-articular (IA) pharmaceutical and anti-nociceptive therapies until operative treatment with arthroplasty is required. Interestingly, there is significant person-to-person variability in the effectiveness of these treatments. However, even in cases in which pharmacologic therapies are effective, their long-term use is associated with numerous potential side effects, including gastric, renal and hepatic damage, addiction and overdose. Therefore, safer, and longer duration therapeutic options are needed to provide relief of pain and functional limitations.

Triamcinolone acetonide extended-release (TA-ER) is an extended-release preparation of triamcinolone acetonide that is U.S. Food and Drug Administration (FDA) approved for treatment of knee pain due to OA. In previous clinical trials, TA-ER provided a predictable release of triamcinolone acetonide as the biocompatible degraded poly-lactic-co-glycolic acid (PLGA) microspheres. A single IA dose of TA-ER resulted in less systemically bioavailable triamcinolone acetonide when compared to a matched dose of triamcinolone acetonide crystalline suspension (TAcs) [1] due to slower release and absorption of TA-ER into the systemic circulation. The more prolonged local exposure to triamcinolone acetonide with TA-ER provided superior analgesic efficacy for knee OA than placebo, with a more significant reduction in average daily pain (ADP) 12 weeks following injection. TA-ER performed more favorably than did TAcs in Western Ontarior and McMaster Universities Arthritis Index (WOMAC) subscale scores for pain, stiffness, and physical function and the KOOS-QOL subscale score at 4, 8, and 12 weeks, but did not provide significant reduction in ADP during this period [2].

Given the incomplete association between reduction in pain and functional limitations, the primary aim of this study was to evaluate the effects of an IA injection of TA-ER on Osteoarthritis Research Society International (OARSI) recommended physical performance measures (30-s chair stand time, 40 ​m fast-paced walking time, stair negotiation time) [3] in adults with bilateral knee OA. Secondarily, this study evaluated patient-reported physical function (KOOS-PS) and quality of life (KOOS-QoL) 6–24 weeks following injection of TA-ER for bilateral symptomatic knee OA.

## 2 Methods

### 2.1 Participants

A total of 70 participants were recruited. Inclusion criteria were age >30 years, bilateral Kellgren-Lawrence (KL) Grade ≥2 with pain in both knees rated ≥4/10 in severity on most days over the prior month, bilateral knee symptoms for ≥3 months, Body mass index (BMI) ​≤ ​41 ​kg/m^2^, able to complete at least 2 of the 3 physical performance tests and abstain from protocol-restricted medications during the study. Exclusion criteria included uncontrolled gout, pseudogout, cancer, hemoglobin (Hgb) A1c>7.2%, other medical conditions more limiting than symptomatic knee OA, recent knee injection (within 12 weeks) or surgery, autoimmune, crystalline, or infectious disease contributing to knee pain, untreated knee injury or clinically significant cognitive impairment or other condition which could compromise participant safety.

Participants were recruited through advertisements in the University of Kansas Medical Center clinics, medical center hallways and websites and in a local newspaper. Lists of patients who had a diagnosis of knee OA in our institutional Healthcare Enterprise Repository for Ontological Narration (HERON) database and agreed to be contacted for studies were contacted and screened for inclusion and exclusion criteria. Qualifying volunteers completed an institutional review board-approved informed consent process (IRB Study#00142926), culminating in signing a consent document in compliance with the Helsinki Declaration. This study was registered at clinicaltrials.gov as NCT03895840.

### 2.2 Study design

In this single-site, open-label study of participants with bilateral knee OA, participants received an IA TA-ER injections in both knees. The purpose of this study was to determine the functional effects of IA injection of TA-ER into both the right and left knees of participants with Kellgren-Lawrence (KL) grade 2–4 symptomatic knee OA [4]. Osteoarthritis Research Society International (OARSI) recommendations for conduct of clinical trials for knee OA were followed [5], and the primary outcome measures were the OARSI-recommended physical performance measures [3]. Patient-reported knee injury and osteoarthritis outcome score physical function (KOOS-PS), knee injury and osteoarthritis outcome score quality of life (KOOS-QoL), and pain were measured at baseline, 6, 12, and 24 weeks after bilateral knee injections to allow assessment of short and long-term effects, consistent with OARSI and Outcome Measures in Rheumatology (OMERACT) recommendations [3,6].

### 2.3 Assessment of knee osteoarthritis

Radiographic knee OA, defined by tibiofemoral KL grade≥2 in both knees [4], was assessed on either knee radiographs acquired within the prior 2 years provided by participants or by coronal plane reconstructions from weight-bearing computed tomography (CT) of the bilateral knee joints (LineUp, CurveBeam, LLC).

### 2.4 Anthropometric measures

Body mass index (BMI, kg/m^2^) was calculated from body mass (kilograms) divided by the square of the participant's height in meters (stadiometer, Holtain, Wales, UK), measured by trained and certified staff [7].

#### 2.4.1 Intervention

A total of 5 ​cc, containing 32 ​mg of TA-ER, an extended-release injectable suspension of triamcinolone acetonide (Zilretta, Flexion Therapeutics, Burlington, MA, USA) was injected into each knee. This formulation is FDA approved as an intra-articular injection for the management of pain due to knee OA. Each injection was prepared according to the manufacturer's recommendations. Sterile chlorhexidine and, per participant preference, a sub-cutaneous skin wheal of 1% lidocaine was provided prior to injection. Continuous sonographic guidance (CX-50, Phillips Healthcare, Amsterdam, Netherlands) was used to identify the superolateral recess of the knee joint capsule in the transverse plane, deep and lateral to the patellar tendon, and instill TA-ER into each knee joint capsule. If greater than 5 ​cc of synovial fluid was visualized, that was aspirated prior to injection of the TA-ER.

#### 2.4.2 Assessment of physical performance (primary outcomes)

The primary outcome was change in physical performance at the 12-week follow-up visit, based on three validated [8] OARSI-recommended performance-based tests for people with knee osteoarthritis [3]. Physical performance was assessed at baseline, 6, 12, and 24 weeks following injections.

#### 2.4.3 30-second chair stand test

The chair stand test evaluated the maximum number of repetitions that participants could stand from a seated position in 30 ​sec [3]. A standardized chair was used for all participants at all visits for which this was recorded. From a seated position, the participant stood up, so the hips and knees achieved the participant's full extension, then completely returned to the seated position. The total number of chair stands (up and down considered one stand) were recorded for the 30-sec period.

#### 2.4.4 Timed stair climb

People with symptomatic knee OA frequently find stair negotiation challenging. Participants were timed ascending and descending a flight of 9 stairs as quickly as they could accomplish in a safe manner (total height of 162 ​cm). Timing began on the signal to start and terminated when the subject returned with both feet to the ground level. Total time was recorded to the nearest hundredth of a second. Use of a handrail and walking aid was permitted if required for safety [3].

#### 2.4.5 40 ​m fast paced walking test

The 40-m fast paced walking test (FPWT) was timed over 4 ​× ​10 ​m (33 ​ft) paths for a total of 40 ​m (132 ​ft) [3]. Sufficient space was provided to turn safely around a cone at each end of the 10 ​m walkway. A practice trial was completed before testing to confirm participant understanding and to minimize learning effects. Participants were timed from their initial crossing of the start line until they crossed that line after completing the course. The stopwatch was paused for turns from the time that participants exited the 10 ​m mark until they re-entered the 10 ​m course. The duration of one trial was recorded to the nearest hundredth of a second and speed was calculated the distance (40 ​m) divided by the time of one test trial (m/s).

#### 2.4.6 Assessment of self-reported outcomes (secondary outcomes)

##### 2.4.6.1 Physical function (KOOS-PS)

In addition to the performance-based primary outcome measures, KOOS-PS, a self-reported measure of mobility was included. The KOOS-Physical Function (KOOS-PS) Short Form is a parsimonious measure of physical function derived from the KOOS [9]. A working group tasked with constructing a composite measure of OA severity sponsored by the Osteoarthritis Research Society International (OARSI) and Outcome Measures in Rheumatology (OMERACT) developed the KOOS-PS. Participants were asked to indicate the degree of difficulty experienced in the last week due to a knee problem in 7 tasks: rising from bed, putting on socks/stockings, rising from sitting, bending to the floor, twisting/pivoting, kneeling, and squatting. Responses were measured on a Likert scale ranging from ‘None’ to ‘Extreme.’

##### 2.4.6.2 KOOS QoL

KOOS-QoL is a 4-question self-reported measure of quality of life, which is part of the five patient-relevant subscales of KOOS [10]. A Likert scale was used with five possible answer options scored from 0 (No Problems) to 4 (Extreme Problems). Scores were transformed to a 0–100 scale, with zero representing extreme knee problems and 100 representing no knee problems.

##### 2.4.6.3 Numerical rating scale (NRS) for pain

The Numerical Rating Scale for Pain (NRS) is a measure of pain intensity. Participants verbally indicated the pain intensity in each knee in response to “On a scale of 0–10, with 0 being no pain at all and 10 being the worst pain imaginable, how would you rate your knee pain in the past 7 days?”

### 2.5 Adverse events

Adverse event information was prospectively collected throughout the duration of each participant's enrollment period and was systematically assessed at the 6, 12, and 24-week follow-up visits. When an adverse event was reported to the study staff, data including the type of event, onset/end dates, duration, severity, and outcome were collected and reported. The principal investigator (NAS) determined the severity of the event using the Common Terminology Criteria for Adverse Events (CTCAE) Version 5.0 guidelines [11].

### 2.6 Statistical analysis

In this open-label study, outcome measures were collected at baseline, 6, 12 and 24 weeks post-treatment. While change in physical performance at 12-week follow-up was the primary outcome, a 24-week follow-up duration was selected to detect meaningful differences and long-term effects, while minimizing the variability that may occur further out from treatment. The overall type I error rate was controlled with an alpha level of 0.0167 to account for three comparisons at the primary time point. Based on the respective minimum detectable change (MDC)_**95**_ calculated from the standard error of the mean change for OARSI recommended physical performance [3], the effect size for this minimal detectible change is 0.392. To detect an effect size at least this large, with an adjusted alpha of 0.0167 and 80% power, a sample size of 60 was required at the 12-week assessment (primary outcome). To accommodate up to 15% dropout, enrollment of 70 participants was planned.

Following acquisition, data were entered into two separate databases by separate members of the study team. The two databases were reconciled with each other, and any discrepancies were reconciled using source documents to confirm data integrity prior to analyses. Continuous variables were summarized with means, standard deviations, minimums and maximums and range checks were completed for data quality assurance. Categorial variables (e.g., sex, KL grade, adverse events) were summarized with frequencies and percentages.

The *a priori* primary analysis regarded the change in the three OARSI-recommended physical performance measures between baseline and 12 weeks following injections. These three pre/post change scores were assessed with one-sample *t*-test for matched pairs, using an alpha level of 0.0167 (0.05/3 tests) to determine statistically significant change. The analyses of secondary and tertiary outcomes involved longitudinal data, collected at baseline, 6, 12, and 24 weeks. These response variables were modeled using the framework of longitudinal mixed models. Each model was based on two main effects: a random subject effect, and a fixed time effect consisting of four levels (baseline and 3 follow-up time points). The Akaike information criterion was used to determine an appropriate variance/covariance structure for each model. Each hypothesis was tested by examining appropriate contrasts and estimated linear forms in the overall mean and the main effects for time. Analyses were participant-based except for NRS, which was knee-based and took into consideration incomplete independence between knees within participants. Analyses were conducted using PROC MIXED in SAS Version 9.4 (SAS Institute, Cary, NC, USA).

## 3 Results

### 3.1 Participant characteristics

**Fig. 1:**
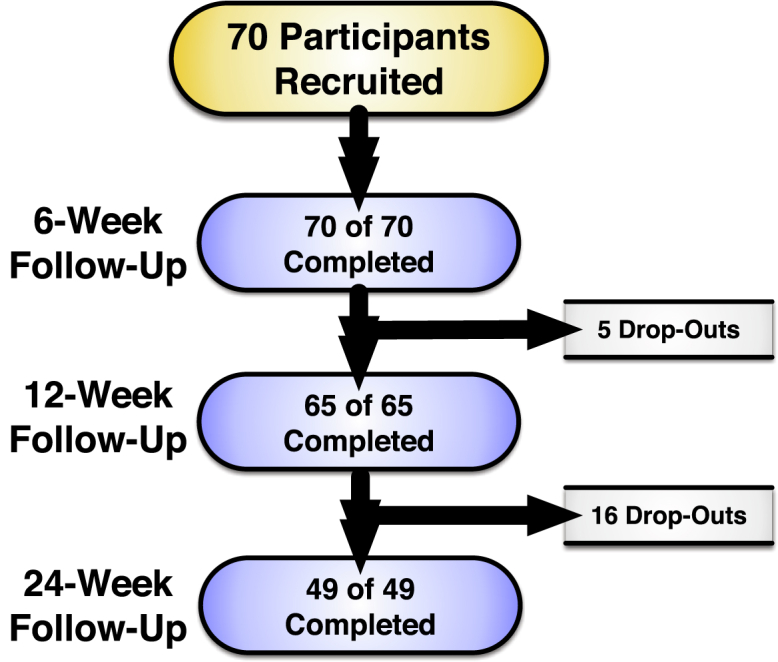
Subject flowchart.

Seventy participants (61.4% women) with a mean ​± ​SD age of 64.0 ​± ​11.7 years and BMI of 31.8 ​± ​5.7 ​kg/m^2^ were enrolled. The demographic and clinical characteristics of this sample population are presented in Table 1. All participants returned for 6-week follow-up. Prior to the 12-week follow-up, 5 participants dropped out of the study. Another 16 participants dropped out after the 12-week visit. A total of 49 participants (70%) completed the 24-week follow-up visit. Fig. 1 details participant flow through the study. Due to precautions relating to the COVID-19 pandemic, 3 participants presented by remote synchronous video at the 6- and 12-week follow-up visits and 2 participants had such visits at the 24-week follow-up.

**Table 1:** Participant clinical characteristics.

Characteristic (Mean ​± ​SD or %)	All (N ​= ​70)	Drop-Outs by Week 24 (N ​= ​21)
Age (years)	64.0 ​± ​11.7	66.0 ​± ​9.2
Sex (% women)	61.3%	61.9%
Body Mass Index (kg/m^2^)	31.8 ​± ​5.7	32.7 ​± ​6.4
Chair stand (#)	7.7 ​± ​3.6	8.6 ​± ​3.4
Stair negotiation (sec)	22.1 ​± ​28.9	22.0 ​± ​13.2
40 ​m FPWT (sec)	37.3 ​± ​26.0	36.6 ​± ​14.8
Physical Function	50.9 ​± ​11.3	46.4 ​± ​9.4
Quality of Life	27.4 ​± ​16.8	20.5 ​± ​15.1

40 ​m FPWT – 40-m fast paced walk test; sec – seconds; kg/m^2^ – kilograms per meter squared.

### 3.2 Functional performance

Baseline and follow-up measurement values, as well as a comparison between baseline and each follow-up time point, controlling for repeated measurements, are presented in Table 2.

**Table 2:** Outcome measures at each follow-up visit (LSMean±SE p-values for comparison with baseline values).

Variables	Baseline	6 weeks	p-value	12 weeks	p-value	24 weeks	p-value
Chair stand (#)	7.7 ​± ​0.4	9.6 ​± ​0.5	**0.0048**	9.5 ​± ​0.5	**0.0111**	8.3 ​± ​0.6	0.4711
Stair negotiation time (sec)	22.1 ​± ​3.5	14.9 ​± ​1.2	0.0530	15.0 ​± ​1.4	0.0599	16.7 ​± ​2.0	0.1793
40 ​m FPWT (sec)	37.3 ​± ​3.1	35.9 ​± ​3.0	0.1758	35.9 ​± ​2.6	0.1560	41.9 ​± ​4.0	**0.0005**
Physical Function	50.9 ​± ​1.4	75.9 ​± ​1.7	**0.0001**	70.0 ​± ​2.1	**0.0001**	56.2 ​± ​2.4	0.0539
Quality of Life	27.4 ​± ​2.0	60.9 ​± ​2.8	**0.0001**	51.9 ​± ​3.4	**0.0001**	37.8 ​± ​3.4	**0.0096**
Knee Pain (NRS)	6.7 ​± ​0.2	2.0 ​± ​0.3	**<0.0001**	3.3 ​± ​0.3	**<0.0001**	5.6 ​± ​0.3	**0.0011**

LSMean – Least Squares Mean; SE – standard error; sec-seconds; NRS- numeric rating scale.

In comparison with baseline values, the number of chair-stands within 30 ​sec significantly improved following treatment by (estimate ±SE) 1.9 ​± ​0.6 ​at 6-week (p ​= ​0.0048) and by 1.8 ​± ​0.5 ​at 12-week follow-up (p ​= ​0.0011), while not statistically significant at 24-week follow-up (0.6 ​± ​0.6 greater chair stands; p ​= ​0.4711). In comparison with baseline values, stair times decreased by 7.2 ​± ​3.7, 7.1 ​± ​3.8, and 5.4 ​± ​4.0 ​sec at the three respective follow-up timepoints, although these changes did not reach statistical significance (p ​= ​0.0530, p ​= ​0.0599, and p ​= ​0.1793 respectively). Of note, while 69 of the 70 participants were able to complete the stair negotiation test at baseline, only 63, 56 and 42 participants were able to complete this test at 6-, 12- and 24-week follow-up, reducing the available sample size for this outcome measurement. In comparison with baseline values, the time for completion of the 40m-FPWT did not significantly change at six weeks (−1.4 ​± ​3.0 ​sec; p ​= ​0.1758) or 12 weeks (−1.4 ​± ​2.6 ​sec; p ​= ​0.1560), while increasing at 24-week follow-up (4.6 ​± ​4.0 sec; p ​= ​0.0005).

### 3.3 Self-reported physical function and quality of life

Self-reported physical function on the KOOS-PS instrument improved in comparison with baseline values at 6- and 12-week follow-up by 25.0 ​± ​1.2 points and 19.1 ​± ​2.1 points, respectively (p ​< ​0.0001), while improvement did not remain statistically significant at 24-week follow-up (5.3 ​± ​2.4 points; p ​= ​0.0539). KOOS-QOL values improved in comparison to baseline values at 6- and 12-week follow-up by 33.5 ​± ​2.8 points and 24.5 ​± ​3.4 respectively (both p ​< ​0.0001), as well as at by 10.4 ​± ​3.4 points at 24-week follow-up (p ​= ​0.0096).

### 3.4 Knee pain

In comparison with baseline values, knee pain NRS was reduced at 6-, 12-, and 24-week follow-up by 4.7 ​± ​0.3 points, 3.4 ​± ​0.3 points (p ​< ​0.0001) and 1.1 ​± ​0.3 points (p ​= ​0.0011) respectively.

#### 3.4.1 Assessment of adverse events

Thirty-four adverse events were reported by 20 of the 70 participants (28.6%), as detailed in Table 3. Most adverse events were expected (19/34 were mentioned in the consent form) and either unrelated (6 events) or unlikely to be related (22 events) to the intervention. Other adverse events were deemed to be possibly related (4 events) or probably related (2 events). The events considered to be probably related were 1) a quarter size bruise the day after the injection, which resolved over 3–4 days, and 2) hyperglycemia in a diabetic participant, peaking at 220 ​mg/dl 4 days following the TA-ER intra-articular injections without observable health effects before returning to the participant's usual level. Falls due to various reasons and knee pain recurrence accounted for greater than one-third of the events. Upper respiratory tract infections, local swelling, headache, and flu-like symptoms a month after the injections comprised another quarter of the adverse events. Most adverse events were mild or moderate in intensity. No serious adverse events occurred during the study.

**Table 3:** Frequencies of adverse events.

Allergic rhinitis	1	Headache	2
Arthralgia	7	Hematuria	1
Blister	1	Hyperglycemia	1
Bruising	3	Otitis Media	1
Epistaxis	1	Rhinorrhea	1
Fall	8	Swelling	2
Flu-Like Symptoms	1	Upper Respiratory Infection	4
		TOTAL	34

Notes: Arthralgia was pain that exceeded the baseline pain severity. Rhinorrhea was differentiated from allergic rhinitis by the color and absence of response to allergy medication.

## 4 Discussion

The primary aim of this study was to evaluate the effects of intra-articular injection of TA-ER on OARSI-recommended physical performance measures (30-sec chair stand test, stair negotiation time, and 40 ​m fast-paced walking time) in adults with bilateral symptomatic knee OA. Secondarily, this study evaluated self-reported physical function (KOOS-PS), quality of life (KOOS-QoL) and knee pain (NRS) 6, 12 and 24 weeks following bilateral knee intra-articular injections with TA-ER. The results provided evidence that a single IA injection of TA-ER into each knee resulted in significant improvements in chair stand performance 6- and 12-weeks following treatment in comparison with baseline. Stair negotiation times were lower at the three respective follow-up timepoints, but were not statistically significant, possibly due to an insufficient sample size for this outcome measurement at follow-up. The time for completion of the 40 ​m FPWT did not significantly improve at follow-up. Self-reported physical function on the KOOS-PS instrument and KOOS-QOL values were improved at 6-and 12-week follow-up, but improvements were attenuated 24 weeks following injections. Knee pain was reduced at all three follow-up time points in comparison with prior to the injections.

Knee OA involves complex interactions between atrophic and proliferative processes that affect all joint tissues with secondary components of inflammation [12]. Intra-articular corticosteroid injections are very common in the treatment of symptomatic knee OA. A randomized, controlled study by Raynauld et al. comparing the effects of IA injection of 40 ​mg triamcinolone acetonide vs. saline every 3 months for up to 2 years in patients with knee OA [12]. In this study, long-term use was not found to result in differences in structural progression, radiographically measured joint space, but did reveal a significant decrease in symptoms in the IA corticosteroid group in comparison with the placebo group [12]. While intra-articular corticosteroid injections are considered to be safe with regard to systemic side effects, in a meta-analysis focusing on the effects on human chondrocytes in vitro and animal articular cartilage in vivo, authors concluded that corticosteroids have a chondrotoxic effect and were associated with greater loss of cartilage volume compared with placebo [13]. Another recent report indicated that in rare cases, IA corticosteroid use could be associated with acceleration of OA progression, rapidly progressive OA with bone loss or subchondral insufficiency fractures [14]. The incidence of such episodes is very low and the etiopathology remains unknown. Furthermore, there is conflicting evidence in vitro, suggesting that low-dose triamcinolone acetonide may confer a protective effect on cartilage, protecting against matrix degradation [15].

Despite the controversy regarding possible chondrotoxic effects of immediate-release intra-articular corticosteroids, the functional improvement detected with TA-ER in the current study and decreased pain following treatment with TA-ER in prior work [2,16] support the need for considering for which patients and where in the knee OA treatment paradigm TA-ER may be indicated to balance potential risks and benefits. This need also is supported by the high level of use in clinical practice (100% of hip and knee surgeon respondents report corticosteroid use and 88.4% indicated always or often using this therapy for treatment of knee OA) [17], and the vast majority of clinical practice guidelines recommend use of intra-articular corticosteroids for knee OA [18], including current guidelines that TA-ER “be used over immediate release to improve patient outcomes” [19].

The current study builds upon prior research that focused mainly on pain relief and contributed additional evidence regarding objective and subjective functional outcomes. Conaghan et al. reported the effects of unilateral injection of TA-ER on average daily pain, Western Ontario and McMaster Universities Osteoarthritis Index (WOMAC) and KOOS-QOL subscale scores, for symptomatic mild-to-moderate knee OA (KL 2–3) in participants ≥40 years of age, but did not focus on functional outcomes [2]. Despite the older age and less severe radiographic grades in the prior study [2], both that study and the current one demonstrated relatively similar effects on the KOOS-QOL subscale. The prior report indicated least squares (LS) Mean ​± ​SE improvement in 12-week KOOS-QOL of 21 ​± ​2 points following unilateral treatment, which is consistent with our study demonstrating improvement of 24.5 ​± ​3.4 points on this scale 12 weeks following bilateral treatment with TA-ER.

One of the strengths of the current study is that we not only evaluated patient reported outcomes of pain and health-related QOL, but also included performance-based measurements. Additionally, this study had wider inclusion criteria for age, BMI, and OA severity as well as including bilateral symptomatic knee OA, allowing generalization of findings to a wider range of patients with bilateral KL 2–4 symptomatic knee OA. Another strength of the current study was the standardization of the procedure and use of ultrasound guidance to visually confirm intra-articular injection.

Limitations of the current study include the absence of a control group preventing comparison of outcomes to other therapeutic options and the higher than anticipated rate of dropout between the 12-week primary outcome and the extended 24-week follow-up. Another limitation resulted from the COVID-19 pandemic requiring us to perform a small number of follow-up visits virtually— three at 6 weeks, three at 12 weeks, and two at 24 weeks (8 out of 184 visits). While the sample size recruited and retained provided sufficient statistical power to assess the OARSI-recommended tests of physical performance [3] at the 12-week primary assessment, the inability of some participants to complete the stair negotiation test reduced statistical power to detect change in this outcome. Specifically, while 60 participants were required to power this outcome, in this study of people with bilateral symptomatic knee OA, not all participants were able to complete the stair negotiation test (69/70 ​at baseline, 63/70 ​at 6 weeks, 56/65 ​at 12 weeks and 42/49 ​at 24 weeks). Participants who dropped out, generally did so after the 12-week visit (16 of 21). Of note, the most common reason to drop out was recurrence of knee pain and many of those who dropped out did so for treatment of their knee pain. Nine of the 16 study participants that dropped out after their 12-week visit did so to have repeat TA-ER injections. Two dropped out between 15 and 16 weeks to undergo cryoneurolysis. Three participants dropped out between 15 and 22 weeks for platelet-rich plasma injections. One participant ended the study at 9 weeks to receive a TA-ER injection due to return of knee pain. The remainder of the participants dropped from the study for varying reasons. One participant requested greater financial incentive at the 24-week visit than was provided per protocol and when not provided, the participant chose not to attend the 24-week follow-up visit. Regarding other participants, three declined to return without providing a reason, one decided to transfer care to another facility, and one reported increased pain at 8 weeks and dropped out.

Future research could advance knowledge regarding potential indications, contraindications, and effects of TA-ER through a comparative effectiveness design and inclusion of different subsets of the knee OA population. The current study was a single-site, open-label study without comparison to legacy therapeutics; further investigation using a randomized, double-blinded, controlled design could further evaluate effects of TA-ER on functional outcomes. Furthermore, it would be useful to investigate the effects of the TA-ER with and without concurrent use of other therapies (e.g. physical therapy or bracing). To date, studies have focused on people with a body mass index <41 ​kg/m^2^ or who have a HgbA1c <7.5%. Though, there is one study by Russell et al. that was a small double blinded, randomized, parallel-group study with 33 patients who had knee OA and a HgbA1c of 6.5–9.0%. That study used continuous glucose monitoring and demonstrated minimal blood glucose disruption with intra-articular TA-ER [20]. There does need to be further research on blood glucose levels as our study detected one episode of hyperglycemia over 200 ​mg/dl at 4 days post-injection. This contrasted with the report by Russell et al. that indicated no serum glucose levels over 180 ​mg/dl past the first 58 ​h and did a single knee injection compared to our bilateral knee injections with a total of 64 ​mg TA-ER being utilized in each patient. Whether this participant had serum glucose levels more sensitive to change or whether this event related to bilateral injections or if it related to external factors not measured in this study is unclear. Future studies could advance knowledge regarding the effects of TA-ER in people with such characteristics to better inform decisions regarding therapeutic risk and benefits and a comparison group would be useful to evaluate if adverse events with TA-ER exceed the background rate for people with knee OA (e.g. frequency of falls).

In conclusion, the findings from this study provided evidence of clinically meaningful and statistically significant improvement in physical performance and self-reported physical function, as well as quality of life 6 and 12 weeks after bilateral TA-ER injections and prolonged improvement in pain and quality of life for up to 24 weeks in adults with bilateral symptomatic knee OA. This was found even in those with end-stage knee OA (KL4). TA-ER demonstrated an acceptable safety profile, with no serious adverse events and most adverse events being mild to moderate. The potential for less systemic effects with TA-ER in comparison with immediate-release triamcinolone acetonide, along with evidence of reduced pain and improved physical function and performance supports a role for TA-ER in the care of adults with symptomatic knee OA.

## Author statement

NA Segal: Conceptualization, Data curation, Formal analysis, Funding acquisition, Investigation, Methodology, Project administration, Resources, Supervision, Writing – original draft, Writing – review & editing. JC Alm: Data curation, Formal analysis, Investigation, Writing – original draft, Writing – review & editing.

## Funding Sources

Funding for this study was provided by Flexion Therapeutics, Inc., a wholly owned subsidiary of Pacira BioSciences, Inc. through an Investigator-Initiated Research (IIR) unrestricted grant (2018-IIR-000006) to the investigators’ institution. The sponsor was not involved in the study design, data collection, data analysis, data interpretation or manuscript preparation. HERON is supported in part by funds from 10.13039/100016220CTSA Award #UL1TR000001 and 10.13039/100006093Patient-Centered Outcomes Research Institute (PCORI) Program Award #CDRN-1306-04631. The authors do not have financial disclosures related to the current study. Unrelated to the current work, Dr. Segal is a consultant for Tenex Health and Integra BioLife.

## Declaration of competing interest

All authors must disclose any financial and personal relationships with other people or organisations that could inappropriately influence (bias) their work. Examples of potential conflicts of interest include employment, consultancies, stock ownership, honoraria, paid expert testimony, patent applications/registrations, and research grants or other funding.

## References

[1] Kraus V.B., Conaghan P.G., Aazami H.A., Mehra P., Kivitz A.J., Lufkin J. (2018). Synovial and systemic pharmacokinetics (PK) of triamcinolone acetonide (TA) following intra-articular (IA) injection of an extended-release microsphere-based formulation (FX006) or standard crystalline suspension in patients with knee osteoarthritis (OA). Osteoarthritis Cartilage.

[2] Conaghan P.G., Hunter D.J., Cohen S.B., Kraus V.B., Berenbaum F., Lieberman J.R. (2018). Effects of a single intra-articular injection of a microsphere formulation of triamcinolone acetonide on knee osteoarthritis pain: a double-blinded, randomized, placebo-controlled, multinational study. J Bone Joint Surg Am.

[3] Dobson F., Hinman R.S., Roos E.M., Abbott J.H., Stratford P., Davis A.M. (2013). OARSI recommended performance-based tests to assess physical function in people diagnosed with hip or knee osteoarthritis. Osteoarthritis Cartilage.

[4] Kellgren J.H., Lawrence J.S. (1957). Radiological assessment of osteo-arthrosis. Ann. Rheum. Dis..

[5] McAlindon T.E., Driban J.B., Henrotin Y., Hunter D.J., Jiang G.L., Skou S.T. (2015). OARSI Clinical Trials Recommendations: design, conduct, and reporting of clinical trials for knee osteoarthritis. Osteoarthritis Cartilage.

[6] Perruccio A.V., Stefan Lohmander L., Canizares M., Tennant A., Hawker G.A., Conaghan P.G. (2008). The development of a short measure of physical function for knee OA KOOS-Physical Function Shortform (KOOS-PS) - an OARSI/OMERACT initiative. Osteoarthritis and cartilage/OARS. Osteoarthritis Research Society.

[7] Segal N.A., Felson D.T., Torner J.C., Zhu Y., Curtis J.R., Niu J. (2007). Greater trochanteric pain syndrome: epidemiology and associated factors. Arch. Phys. Med. Rehabil..

[8] Dobson F., Hinman R.S., Hall M., Marshall C.J., Sayer T., Anderson C. (2017). Reliability and measurement error of the Osteoarthritis Research Society International (OARSI) recommended performance-based tests of physical function in people with hip and knee osteoarthritis. Osteoarthritis Cartilage.

[9] Davis A.M., Perruccio A.V., Canizares M., Hawker G.A., Roos E.M., Maillefert J.F. (2009). Comparative, validity and responsiveness of the HOOS-PS and KOOS-PS to the WOMAC physical function subscale in total joint replacement for osteoarthritis. Osteoarthritis Cartilage.

[10] Gandek B., Ware J.E. (2017). Validity and responsiveness of the knee injury and osteoarthritis outcome score: a comparative study among total knee replacement patients. Arthritis Care Res..

[11] Freites-Martinez A., Santana N., Arias-Santiago S., Viera A. (2021). Using the common Terminology criteria for adverse events (CTCAE - version 5.0) to evaluate the severity of adverse events of anticancer therapies. Actas Dermosifiliogr (Engl Ed).

[12] Raynauld J.P., Buckland-Wright C., Ward R., Choquette D., Haraoui B., Martel-Pelletier J. (2003). Safety and efficacy of long-term intraarticular steroid injections in osteoarthritis of the knee: a randomized, double-blind, placebo-controlled trial. Arthritis Rheum..

[13] Kompel A.J., Roemer F.W., Murakami A.M., Diaz L.E., Crema M.D., Guermazi A. (2019). Intra-articular corticosteroid injections in the hip and knee: perhaps not as safe as we thought?. Radiology.

[14] Guermazi A., Neogi T., Katz J.N., Kwoh C.K., Conaghan P.G., Felson D.T. (2020). Intra-articular corticosteroid injections for the treatment of hip and knee osteoarthritis-related pain: considerations and controversies with a focus on imaging-radiology scientific expert panel. Radiology.

[15] Frank E., Hung H.H., Krishnan Y., Senter B., Bodick N., Grodzinsky A. (2019). Dose-dependent chondroprotective effects of triamcinolone acetonide on inflamed and injured cartilage using an in vitro model. Osteoarthritis Cartilage.

[16] Conaghan P.G., Cohen S.B., Berenbaum F., Lufkin J., Johnson J.R., Bodick N. (2018). Brief report: a phase IIb trial of a novel extended-release microsphere formulation of triamcinolone acetonide for intraarticular injection in knee osteoarthritis. Arthritis Rheumatol..

[17] Blankstein M., Lentine B., Nelms N.J. (2021). Common practices in intra-articular corticosteroid injection for the treatment of knee osteoarthritis: a survey of the American association of hip and knee surgeons membership. J. Arthroplasty.

[18] Phillips M., Bhandari M., Grant J., Bedi A., Trojian T., Johnson A. (2021). A systematic review of current clinical practice guidelines on intra-articular hyaluronic acid, corticosteroid, and platelet-rich plasma injection for knee osteoarthritis: an international perspective. Orthop J Sports Med.

[19] Management of Osteoarthritis of the Knee (Non- Arthroplasty) Evidence-Based Clinical Practice Guideline. https://www.aaos.org/oak3cpg ed: American Academy of Orthopaedic Surgeons Board of Directors 2021:58. Accessed November 3, 2021.

[20] Russell S.J., Sala R., Conaghan P.G., Habib G., Vo Q., Manning R. (2018). Triamcinolone acetonide extended-release in patients with osteoarthritis and type 2 diabetes: a randomized, phase 2 study. Rheumatology.

